# Carbon tetrachloride induced kidney and lung tissue damages and antioxidant activities of the aqueous rhizome extract of *Podophyllum hexandrum*

**DOI:** 10.1186/1472-6882-11-17

**Published:** 2011-02-28

**Authors:** Showkat Ahmad Ganie, Ehtishamul Haq, Abid Hamid, Yasrib Qurishi, Zahid Mahmood, Bilal Ahmad Zargar, Akbar Masood, Mohmmad Afzal Zargar

**Affiliations:** 1 Department of Biochemistry, University of Kashmir, Srinagar, 190006, India; 2 Department of Pharmaceutical Sciences, University of Kashmir, Srinagar, 190006, India; 3 Department of Biotechnology, University of Kashmir, Srinagar, 190006, India; 4 Indian Institute of Integrative Medicine (Council of Scientific & Industrial Research), Jammu, India

## Abstract

**Background:**

The present study was conducted to evaluate the *in vitro *and *in vivo *antioxidant properties of aqueous extract of *Podophyllum hexandrum*. The antioxidant potential of the plant extract under *in vitro *situations was evaluated by using two separate methods, inhibition of superoxide radical and hydrogen peroxide radical. Carbon tetrachloride (CCl_4_) is a well known toxicant and exposure to this chemical is known to induce oxidative stress and causes tissue damage by the formation of free radicals.

**Methods:**

36 albino rats were divided into six groups of 6 animals each, all animals were allowed food and water *ad libitum*. Group I (control) was given olive oil, while the rest groups were injected intraperitoneally with a single dose of CCl_4 _(1 ml/kg) as a 50% (v/v) solution in olive oil. Group II received CCl_4 _only. Group III animals received vitamin E at a concentration of 50 mg/kg body weight and animals of groups IV, V and VI were given extract of *Podophyllum hexandrum *at concentration dose of 20, 30 and 50 mg/kg body weight. Antioxidant status in both kidney and lung tissues were estimated by determining the activities of antioxidative enzymes, glutathione reductase (GR), glutathione peroxidase (GPX), glutathione-S-transferase (GST) and superoxide dismutase (SOD); as well as by determining the levels of reduced glutathione (GSH) and thiobarbituric acid reactive substances (TBARS). In addition, superoxide and hydrogen peroxide radical scavenging activity of the extract was also determined.

**Results:**

Results showed that the extract possessed strong superoxide and hydrogen peroxide radical scavenging activity comparable to that of known antioxidant butylated hydroxy toluene (BHT). Our results also showed that CCl_4 _caused a marked increase in TBARS levels whereas GSH, SOD, GR, GPX and GST levels were decreased in kidney and lung tissue homogenates of CCl_4 _treated rats. Aqueous extract of *Podophyllum hexandrum *successfully prevented the alterations of these effects in the experimental animals.

**Conclusion:**

Our study demonstrated that the aqueous extract of *Podophyllum hexandrum *could protect the kidney and lung tissue against CCl_4 _induced oxidative stress probably by increasing antioxidant defense activities.

## Background

Reactive oxygen species, including superoxide radicals (O_2_^•^-), hydrogen peroxide (H_2_O_2_) and hydroxyl radicals (OH^•^) are generated as byproducts of normal metabolism [1,2]. Cumulative oxidative damage leads to numerous diseases and disorders [3]. The enhanced production of free radicals and oxidative stress can also be induced by a variety of factors such as radiation or exposure to heavy metals and xenobiotics (e.g., carbon tetrachloride) [4]. Carbon tetrachloride (CCl_4_) intoxication in animals is an experimental model that mimics oxidative stress in many pathophysiological situations [5]. CCl_4 _intoxication in various studies has demonstrated that CCl_4 _causes free radical generation in many tissues such as liver, kidney, heart, lung, brain and blood [6]. The toxicity of CCl_4 _probably depends on formation of the trichloromethyl radical (CCl_3_^•^), which in the presence of oxygen interacts with it to form the more toxic trichloromethyl peroxyl radical (CCl_3_O_2_^•^) [7]. Studies also showed that various herbal extracts could protect organs against CCl_4 _induced oxidative stress by altering the levels of increased lipid peroxidation and enhancing the decreased activities of antioxidant enzymes, like superoxide dismutase (SOD), catalase (CAT) and glutathione-S-transferase (GST) as well as enhanced the decreased level of the reduced glutathione (GSH) [8]. In the modern medicine, plants occupy a significant berth as raw materials for some important drug preparations [9]. *Podophyllum hexandrum *(PH) has been extensively exploited in traditional Ayurvedic system of medicine for treatment of a number of ailments like Condyloma acuminata, Taenia capitis, monocytoid leukemia, Hodgkins disease, non-Hodgkin's Lymphoma, cancer of brain, lung, bladder and venereal warts [10]. PH is reported to contain a number of compounds with significant pharmacological properties, e.g. epipodophyllotoxin, podophyllotoxone, 4-methylpodophyllotoxin, aryltetrahydronaphthalene lignans, flavonoids such as quercetin, quercetin-3-glycoside, 4-demethylpodophyllotoxin glycoside, podophyllotoxinglycoside, kaempferol and kaempferol-3-glucoside [11,12]. In this particular study, protective role of aqueous extract of the *Podophyllum hexandrum *was evaluated against free radical mediated damages under *in vitro *and *in vivo *situations. *In vitro *assays were carried on superoxide radical scavenging activity and hydrogen peroxide radical scavenging activity. Here, kidney and lung toxicity was induced by administering a single dose of CCl_4 _into experimental adult male albino rats and radical scavenging activity of the extract was evaluated by measuring the levels of GSH and extent of lipid peroxidation in kidney and lung tissue homogenates and activity of antioxidant enzymes via SOD, GP_X_, GR and GST. In addition, study on the effect of a known antioxidant, vitamin E, was also included against CCl_4 _induced kidney and lung oxidative stress. The major aim of the present study was to examine the protective mechanisms of aqueous extract of PH in kidney and lung tissues in carbon tetrachloride intoxicated rats.

## Methods

### Plant material collection and extraction

The rhizome of *Podophyllum hexandrum *was collected from higher reaches of Aharbal, Shopian, J&K, India in the month of May and June, identified by the Centre of Plant Taxonomy, Department of Botany, University of Kashmir, and authenticated by Dr. Irshad Ahmad Nawchoo (Department of Botany) and Akhter Hussain Malik (Curator, Centre for Plant Taxonomy, University of Kashmir). A reference specimen has been retained in the herbarium of the Department of Botany at the University of Kashmir under reference number KASH- bot/Ku/PH- 702- SAG.

The plant material (rhizome) was dried in the shade at 30 ± 2°C. The dried rhizome material was ground into a powder using mortar and pestle and passed through a sieve of 0.3 mm mesh size. The powder obtained was extracted with water using a Soxhlet extractor (60-80°C). The extract was then concentrated with the help of rotary evaporator under reduced pressure and the solid extract was stored in refrigerator for future use.

### Animals

Adult male albino rats of Wistar strain weighing 200-250 g used throughout this study were purchased from the Indian Institute of Integrative Medicine Jammu (IIIM). The animals had access to food and water *ad libitum*. The animals were maintained in a controlled environment under standard conditions of temperature and humidity with an alternating 12 hr light and dark cycle. The animals were maintained in accordance with the guidelines prescribed by the National Institute of Nutrition, Indian Council of Medical Research and the study was approved by the Animal Ethics Committee of the University of Kashmir.

### Experimental methods

#### Assessment of superoxide anion radical scavenging property

Superoxide anion radical generated by the Xanthine/Xanthine oxidase system was spectrophotometrically determined by monitoring the product of nitroblue tetrazolium (NBT) using the method of Jung [13]. A reaction mixture containing 1.0 ml of 0.05 M phosphate buffer (pH 7.4), 0.04 ml of 0.15% BSA, 0.04 ml of 15.0 mM NBT and various concentrations of plant extract and known antioxidant was incubated at 25°C for 10 min, and the reaction was then started by adding 0.04 ml of 1.5 U/ml Xanthine oxidase and again incubated at 25°C for 20 min. The absorbance of the reaction mixture was measured at 560 nm. Decreased absorbance of the reaction mixture indicates increased superoxide anion radical scavenging activity.

The scavenging activity of the plant extract on Superoxide anion radical was expressed as:

$$\%\text{ inhibition}=[({\text{A}}_{0}-{\text{A}}_{\text{1}})/{\text{A}}_{0}]\times \text{1}00$$

Where A_0 _was the absorbance of the control and A_1 _was absorbance in the presence of *Podophyllum hexandrum *extract/known antioxidant.

#### Assessment of Hydrogen peroxide scavenging activity

The ability of *Podophyllum hexandrum *aqueous extract to scavenge hydrogen peroxide was evaluated according to the method of Ruch [14]. A solution of H_2_O_2 _(2 mmol) was prepared in phosphate buffer (pH 7.5). Plant extract (50-300 μg/ml) was added to the hydrogen peroxide solution (0.6 ml). Absorbance of hydrogen peroxide at 230 nm was determined after 15 minutes against a blank solution containing phosphate buffer without hydrogen peroxide. BHT was taken as known standard. The scavenging activity of the plant extract on H_2_O_2 _was expressed as:

$$\%\text{ scavenged }[{\text{H}}_{\text{2}}{\text{O}}_{\text{2}}]=[({\text{A}}_{0}-{\text{A}}_{\text{1}})/{\text{A}}_{0}]\times \text{1}00$$

Where A_0 _is the absorbance of the control and A_1 _is absorbance in the presence of plant extract and known standard.

### Dosage and treatment

Rats were divided into six groups each containing six rats. The plant extract was employed at oral doses of 20, 30 and 50 mg/kg-day. The extract was suspended in normal saline such that the final volume of extract at each dose was 1 ml which was fed to rats by gavage.

**Group I **- Received olive oil vehicle only at 5 ml/kg-day.

**Group II **- Received CCl_4 _in olive oil vehicle only.

**Group III **- Were administered with vitamin E (50 mg/kg-day).

**Group IV **- Received 20 mg/kg-day extract orally for fifteen days.

**Group V **- Received 30 mg/kg-day extract orally for fifteen days.

**Group VI **- Received 50 mg/kg-day orally for fifteen days.

On the thirteenth day, animals from groups II-VI were injected intraperitoneally with CCl_4 _in olive oil vehicle at a dosage of 1 ml/kg bw. The rats were sacrificed 48 hr after CCl_4 _administration and kidney and lung tissue was isolated out, and post mitochondrial supernatant of both the tissues was prepared.

#### Preparation of post mitochondrial supernatant (PMS)

Kidney and lung tissue was washed in ice-cold 1.15% KCl and homogenized in a homogenizing buffer (50 mM Tris- HCl, 1.15% KCl pH 7.4) using a Teflon homogenizer. The homogenate was centrifuged at 9,000 g for 20 minutes to remove debris. The supernatant was further centrifuged at 15,000 g for 20 minutes at 4°C to get PMS which was used for various biochemical assays. Protein concentration was estimated by the method of Lowry [15].

#### Estimation of lipid peroxidation (PMS)

Lipid peroxidation in tissues was estimated by the formation of thiobarbituric acid reactive substances (TBARS) by the method of Nichans and Samuelson [16]. In brief 0.1 ml of tissue homogenate (PMS; Tris- HCl buffer, pH 7.5) was treated with 2 ml of (1:1:1 ratio) TBA-TCA-HCl reagent (0.37% thiobarbituric acid, 0.25 N HCl, and 15% TCA), placed in boiling water bath for 15 min, cooled and centrifuged at room temperature for 10 min. The absorbance of the clear supernatant was measured against reference blank at 535 nm.

#### Determination of total sulphydryl groups

The acid soluble sulphydryl groups (non protein thiols of which more than 93% is reduced glutathione (GSH) forms a yellow colored complex with DTNB that shows the absorption maximum at 412 nm. The assay procedure will be followed to that of Moren [17]. 500 μl of homogenate precipitated with 100 μl of 25% TCA, will be then subjected to centrifugation at 3000 g for 10 minutes to settle the precipitate. 100 μl of the supernatant obtained shall be added to the test tube containing the 2 ml of 0.6 mM DTNB and 0.9 ml of 0.2 mM sodium phosphate buffer (pH 7.4). The yellow color obtained will be measured at 412 nm against the reagent blank which contains 100 μl of 25% TCA in place of the supernatant. Sulphydryl content shall be calculated using the DTNB molar extension coefficient of 13,100.

#### Glutathione peroxidase (GP_X_)

GP_X _activity was assayed using the method of Sharma [18]. The assay mixture consists of 1.49 ml of sodium phosphate buffer (0.1 M pH 7.4), 0.1 ml EDTA (1 mM), 0.1 ml sodium azide (1 mM), 0.1 ml 1 mM GSH, 0.1 ml of NADPH (0.02 mM), 0.01 ml of 1 mM H_2_O_2 _and 0.1 ml PMS in a total volume of 2 ml. Oxidation of NADPH was recorded spectrophotometrically at 340 nm and the enzyme activity was calculated as nmoles NADPH oxidized/min/mg of protein, using € of 6.22 × 10^3 ^M^-1 ^cm^-1^.

#### Glutathione Reductase activity (GR)

GR activity was assayed by the method of Sharma [18]. The assay mixture consisted of 1.6 ml of sodium phosphate buffer (0.1 M pH 7.4), 0.1 ml EDTA (1 mM), 0.1 ml 1 mM oxidized glutathione, 0.1 ml of NADPH (0.02 mM), 0.01 ml of 1 mM H_2_O_2 _and 0.1 ml PMS in a total volume of 2 ml. The enzyme activity measured at 340 nm was calculated as nmoles of NADPH oxidized/min/mg of protein using € of 6.22 × 10^3 ^M^-1 ^cm^-1^.

#### Glutathione-S-transferase (GST) activity

GST activity was assayed using the method of Haque [19]. The reaction mixture consisted of 1.67 ml sodium phosphate buffer (0.1 M pH 6.5), 0.2 ml of 1 mM GSH, 0.025 ml of 1 mM CDNB and 0.1 ml of PMS in a total volume of 2 ml. The change in absorbance was recorded at 340 nm and the enzyme activity was calculated as nmoles of CDNB conjugates formed/min/mg protein using € of 9.6 × 10^3 ^M^-1 ^cm^-1^.

#### Super oxide dismutase activity (SOD)

SOD activity was estimated by Beauchamp and Fridovich [20]. The reaction mixture consisted of 0.5 ml of hepatic PMS, 1 ml of 50 mM sodium carbonate, 0.4 ml of 25 μM NBT and 0.2 ml of 0.1 mM EDTA. The reaction was initiated by addition of 0.4 ml of 1 mM hydroxylamine-hydrochloride. The change in absorbance was recorded at 560 nm. The control was simultaneously run without tissue homogenate. Units of SOD activity were expressed as the amount of enzyme required to inhibit the reduction of NBT by 50%.

### Statistical analysis

The values are expressed as mean ± standard deviation (SD). The results were evaluated by using the SPSS (version 12.0) and Origin 6 softwares and evaluated by one-way ANOVA followed by Bonferroni t-test. Statistical significance was considered when value of *P *was < 0.5.

## Results

### Superoxide anion radical scavenging activity

Superoxide anion radical scavenging activity of varying amount of aqueous extract of *Podophyllum hexandrum *was determined by Xanthine-Xanthine oxidase system. Table 1 shows the percentage inhibition of superoxide radical generation of 50-300 μg of extract and comparison with the same amount of BHT. The aqueous extract of *Podophyllum hexandrum *exhibited somewhat lesser superoxide radical scavenging activity than BHT. The percentage inhibition of superoxide generation at a concentration of 300 μg/ml of aqueous extract of *Podophyllum hexandrum *and BHT was however found as 81.64 and 85.71%, suggesting that *Podophyllum hexandrum *has strong superoxide radical scavenging activity at higher concentration.

**Table 1:** Effect of *Podophyllum hexandrum *aqueous extract and known antioxidant (BHT) on superoxide radical scavenging activity.

Concentration (μg/ml)	Aqueous extract of P.H	BHT
50 (μg/ml)	17.01 ± 2.08	40.30 ± 3.78

100 (μg/ml)	33.62 ± 0.75	52.16 ± 2.55

150(μg/ml)	47.19 ± 2.55	63.65 ± 2.31

200(μg/ml)	61.44 ± 2.85	71.63 ± 2.2

250(μg/ml)	72.74 ± 3.64	81.64 ± 1.11

300(μg/ml)	81.64 ± 1.11	85.71 ± 1.40

Effect of *Podophyllum hexandrum *aqueous extract and known antioxidant (BHT) on superoxide radical scavenging activity. Absorbance of control at 560 nm = 0.899 ± 0.25. The results represent mean ± S.D of 3 separate experiments.

### Hydrogen peroxide radical scavenging activity

Table 2 shows the scavenging effect of *Podophyllum hexandrum *extract on H_2_O_2 _and the comparison with standard BHT in an amount dependent manner. As shown in table the extract and BHT exhibited 67.51 and 72.17% scavenging activity on hydrogen peroxide at 300 μg/ml respectively, again suggests that *Podophyllum hexandrum *extract possess a strong free radical scavenging activity, comparable to that of BHT.

**Table 2:** Effect of *Podophyllum hexandrum *aqueous extract and known antioxidant (BHT) on hydrogen peroxide radical scavenging activity.

Concentration (μg/ml)	Aqueous extract of P.H	BHT
50 (μg/ml)	22.30 ± 1.20	30.32 ± 1.14

100 (μg/ml)	32.07 ± 4.27	45.86 ± 1.50

150(μg/ml)	43.10 ± 1.15	52.12 ± 1.15

200(μg/ml)	53.45 ± 1.57	58.64 ± 1.98

250(μg/ml)	62.22 ± 2.75	66.66 ± 1.90

300(μg/ml)	67.51 ± 2.02	72.17 ± 1.50

Effect of *Podophyllum hexandrum *aqueous extract and known antioxidant (BHT) on hydrogen peroxide radical scavenging activity. Absorbance of control at 230 nm = 0.665 ± 0.15. The results represent mean ± S.D of 3 separate experiments.

### Effect of aqueous extract on lipid peroxidation in CCl_4 _treated rats

**Figure 1:**
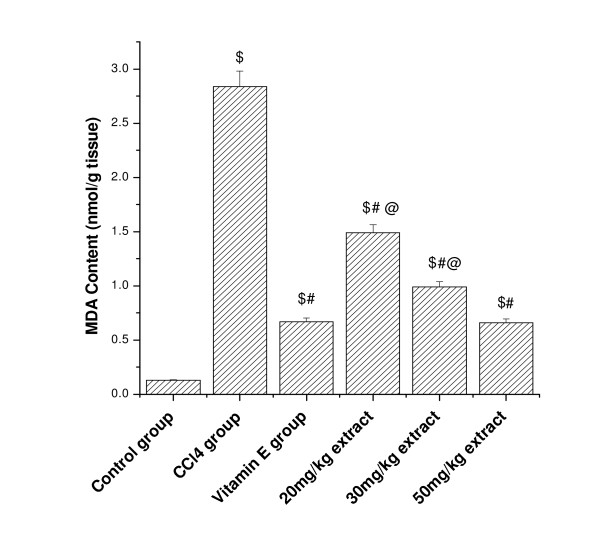
**Represents the effect of aqueous extract on kidney tissue homogenate lipid peroxidation in CCl_4 _treated rats**. $; *p *< 0.001, as compared with normal control group, #; *p *< 0.001 as compared with CCl_4 _group, @; *p *< 0.001 as compared with V.E. The data were presented as means ± SD for six animals in each observation and evaluated by one-way ANOVA followed by Bonferroni t - test to detect inter group differences. Differences were considered to be statistically significant if p < 0.05.

**Figure 2:**
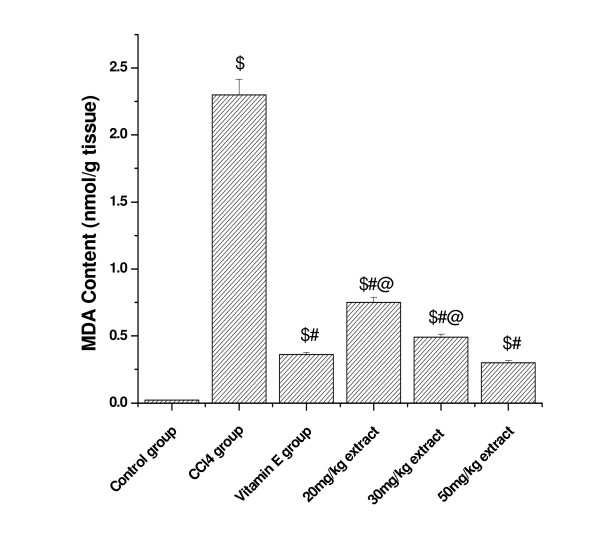
**Represents the effect of aqueous extract on lung tissue homogenate lipid peroxidation in CCl_4 _treated rats**. $; *p *< 0.001, as compared with normal control group, #; *p *< 0.001 as compared with CCl_4 _group, @; *p *< 0.001 as compared with V.E. The data were presented as means ± SD for six animals in each observation and evaluated by one-way ANOVA followed by Bonferroni t - test to detect inter group differences. Differences were considered to be statistically significant if p < 0.05.

TBARS concentrations (expressed as MDA) in the kidney and lung tissue homogenates of all the experimental animals are shown in Figure 1 and 2. After CCl_4 _administration, the MDA levels increased significantly from 0.12 to 2.8 nmol/mg protein in kidney tissue homogenate and in lung tissue homogenate, the MDA level increased from 0.02 to 2.30 nmol/mg protein. However pretreatment of aqueous extract of *Podophyllum hexandrum *for 15 days decreased the MDA level in a dose dependent manner in both tissue homogenates. Vitamin E treated animals also showed significant decrease in the MDA levels as compared to CCl_4 _treated animals.

In order to investigate whether the antioxidant activities of *Podophyllum hexandrum *are mediated by an increase in antioxidant enzymes, we measured GPx, GR, SOD and GST activities in kidney and lung tissues of rats treated with *Podophyllum hexandrum *rhizome aqueous extract. In the present study, treatment of rats with *Podophyllum hexandrum *rhizome aqueous extract significantly increased rat, kidney and lung tissue SOD, GP_X_, GR and GST activities.

### Effect on (GP_X _activity)

**Figure 3:**
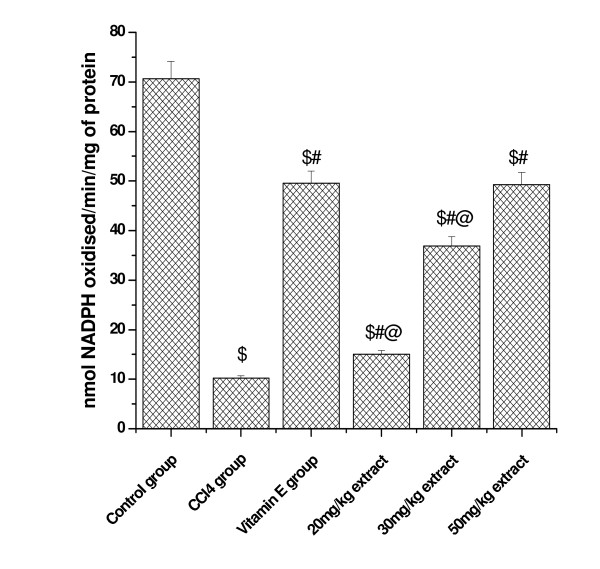
**Represents the dose dependent effect of aqueous extract of *Podophyllum hexandrum *on glutathione peroxidase activity against CCl4 induced toxicity in Kidney tissue homogenate**. $; *p *< 0.001, as compared with normal control group, #; *p *< 0.001 as compared with CCl_4 _group, @; *p *< 0.001 as compared with V.E. The data were presented as means ± SD for six animals in each observation and evaluated by one-way ANOVA followed by Bonferroni t - test to detect inter group differences. Differences were considered to be statistically significant if p < 0.05.

**Figure 4:**
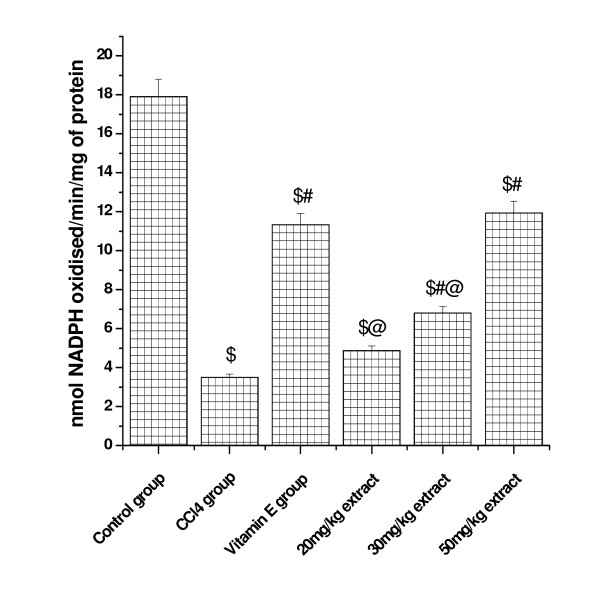
**Represents the dose dependent effect of aqueous extract of *Podophyllum hexandrum *on glutathione peroxidase activity against CCl4 induced toxicity in lung tissue homogenate**. $; *p *< 0.001, as compared with normal control group, #; *p *< 0.001 as compared with CCl_4 _group, @; *p *< 0.001 as compared with V.E. The data were presented as means ± SD for six animals in each observation and evaluated by one-way ANOVA followed by Bonferroni t - test to detect inter group differences. Differences were considered to be statistically significant if p < 0.05.

(Figure 3 and 4) shows that glutathione peroxidase activity in kidney and lung tissue was significantly decreased in CCl_4 _treated animals compared to control. Pretreatment with aqueous extract significantly increased the GP_X _activity in a dose dependent manner. At higher concentrations of plant extract (50 mg/kg dose level), the activity was increased to 49.30 from CCl_4 _treated group (11.20) in kidney tissue and the level was increased to 11.93 from 3.51 at the same concentration in lung tissue. Vitamin E (50 mg/kg) treated animals also showed significant increase in GP_X _activity in both the tested organs.

### Effect on GR activity

**Figure 5:**
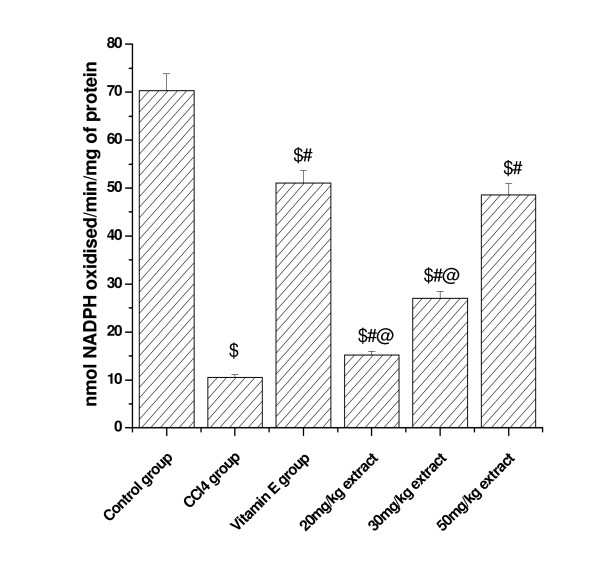
**Represents the effect of aqueous extract of *Podophyllum hexandrum *on the glutathione reductase activity in kidney tissue**. $; *p *< 0.001, as compared with normal control group, #; *p *< 0.001 as compared with CCl_4 _group, @; *p *< 0.001 as compared with V.E. The data were presented as means ± SD for six animals in each observation and evaluated by one-way ANOVA followed by Bonferroni t - test to detect inter group differences. Differences were considered to be statistically significant if p < 0.05.

**Figure 6:**
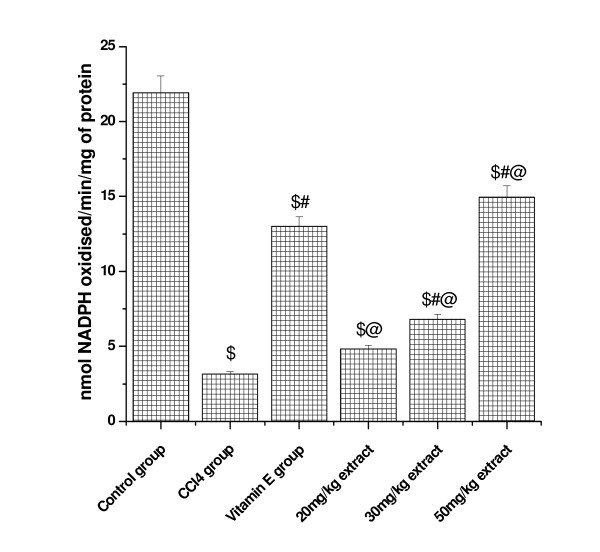
**Represents the effect of aqueous extract of *Podophyllum hexandrum *on the glutathione reductase activity in lung tissue**. $; *p *< 0.001, as compared with normal control group, #; *p *< 0.001 as compared with CCl_4 _group, @; *p *< 0.001 as compared with V.E. The data were presented as means ± SD for six animals in each observation and evaluated by one-way ANOVA followed by Bonferroni t - test to detect inter group differences. Differences were considered to be statistically significant if p < 0.05.

Glutathione reductase (GR) activity was significantly decreased in CCl_4 _treated animals when compared to control group. There was a significant increase in glutathione reductase activity observed in aqueous extract treated groups in both the tested organs. At the higher concentration of plant extract (50 mg/kg) the activity increased many fold (Figure 5 and 6). Similar results were obtained with vitamin E.

### Effect on SOD activity

**Figure 7:**
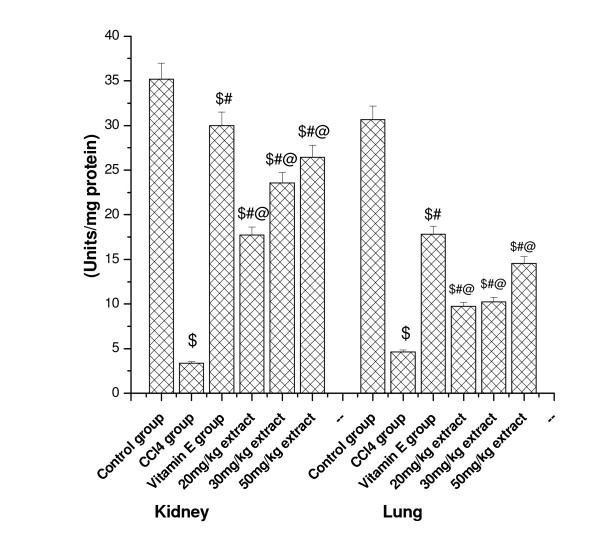
**Represents the effect of aqueous extract of *Podophyllum hexandrum *on the superoxide dismutase activity in kidney and lung tissue organs**. $; *p *< 0.001, as compared with normal control group, #; *p *< 0.001 as compared with CCl_4 _group, @; *p *< 0.001 as compared with V.E. The data were presented as means ± SD for six animals in each observation and evaluated by one-way ANOVA followed by Bonferroni t - test to detect inter group differences. Differences were considered to be statistically significant if p < 0.05.

Effects of CCl_4 _and CCl_4 _plus aqueous extract of *Podophyllum hexandrum *treatments on kidney and lung tissue SOD activity is presented in Figure 7. The SOD activity of both the tested organs significantly decreased in CCl_4 _treated group. Administration of extract proved significantly better in restoring the altered activity of antioxidant enzyme like SOD, and increased the activity in a dose dependent manner in both organs. Similar results were observed in vitamin E treated group.

### Effect on GSH level

**Figure 8:**
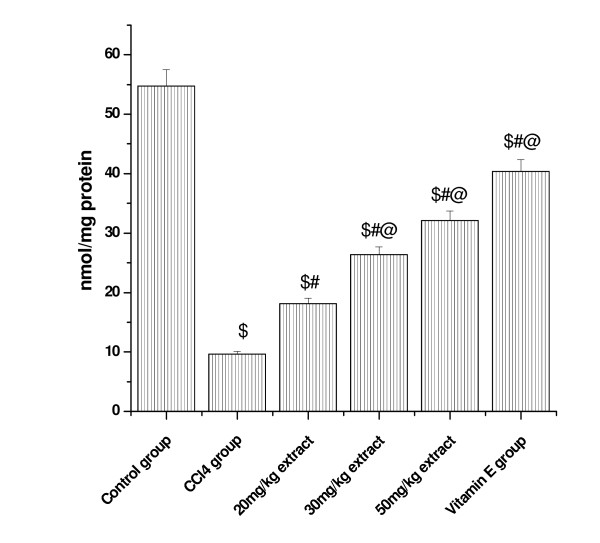
**Represents the effect of aqueous extract on GSH levels in CCl4 induced Kidney damages in rats**. $; *p *< 0.001, as compared with normal control group, #; *p *< 0.001 as compared with CCl_4 _group, @; *p *< 0.001 as compared with V.E. The data were presented as means ± SD for six animals in each observation and evaluated by one-way ANOVA followed by Bonferroni t - test to detect inter group differences. Differences were considered to be statistically significant if p < 0.05.

**Figure 9:**
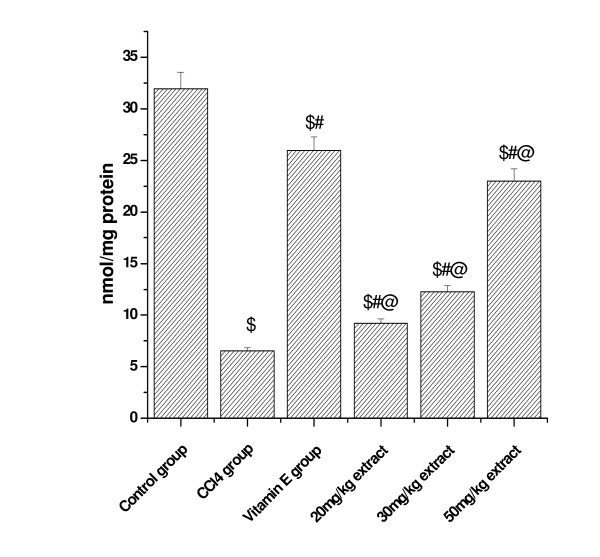
**Represents the effect of aqueous extract of *Podophyllum hexandrum *on GSH levels in CCl4 induced lung damages in rats**. $; p < 0.001, as compared with normal control group, #; p < 0.001 as compared with CCl4 group, @; p < 0.001 as compared with V.E. The data were presented as means ± SD for six animals in each observation and evaluated by one-way ANOVA followed by Bonferroni t - test to detect inter group differences. Differences were considered to be statistically significant if p < 0.05.

CCl_4 _administration markedly decreased the levels of reduced glutathione in both the kidney (control = 54.73 microgram/mg protein) and lung tissue (control = 31.93 microgram/mg protein) to 9.62 (CCl_4 _group kidney) and 6.53 (CCl_4 _group lung) demonstrating oxidative stress. Pretreatment with the aqueous extract of *Podophyllum hexandrum *significantly ameliorated CCl_4_-induced depletion of GSH in both lung and kidney in a dose dependent manner (Figure 8 and 9).

### Effect on GST activity

**Figure 10:**
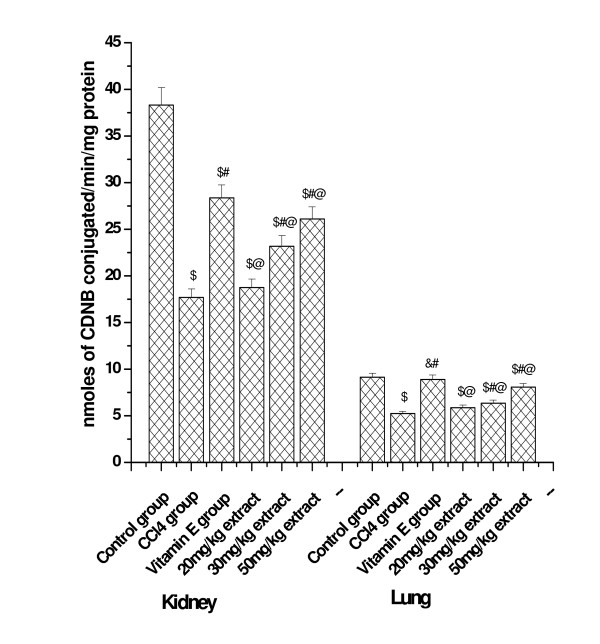
**Effect of aqueous extract on glutathione-S-transferase activity**. Left panel shows the effect of extract on kidney tissue and right panel shows the effect on lung tissue against CCl4 induced damages. $; p < 0.001, as compared with normal control group, #; p < 0.001 as compared with CCl4 group, @; p < 0.001 as compared with V.E; &; non significant as compared with normal control. The data were presented as means ± SD for six animals in each observation and evaluated by one-way ANOVA followed by Bonferroni t - test to detect inter group differences. Differences were considered to be statistically significant if p < 0.05.

GST activity as measured from the lung and kidney tissue homogenates of all the experimental animals have been shown in Figure 10. In the kidney tissue homogenate decreased GST activity was observed in CCl_4 _treated animals (17.71 nmoles) compared to the normal control group (38.28 nmoles). Pretreatment with the aqueous extract for 15 days prior to CCl_4 _intoxication enhanced that activity significantly in a dose dependent manner. In the lung homogenate GST activity of CCl_4 _treated group (5.22 nmoles) was lower compared to that in the normal group (9.11 nmoles), while the GST activity was found to be increased in the lung tissue homogenate of rats treated with aqueous extract at the concentration of 20 and 30 mg/kg bw for 15 days prior to CCl_4 _treatment. GST activity in vitamin E and 50 mg/kg bw plant extract pretreated group was close to the normal level in lung tissue.

## Discussion

CCl_4 _when administrated is distributed and deposited to organs such as the liver, brain, kidney, lung and heart [21]. The reactive metabolite trichloromethyl radical (^•^CCl3) and trichloromethyl peroxide radical (CCl_3_O_2_•) has been formed from the metabolic conversion of CCl_4 _by cytochrome P-450. As O_2 _tension rises, a greater fraction of ^•^CCl_3 _present in the system reacts very rapidly with O_2 _and more reactive free radicals, like CCl_3_OO^• ^is generated from ^•^CCl_3_. These free radicals initiate the peroxidation of membrane poly unsaturated fatty acids (PUFA), cell necrosis, GSH depletion, membrane damage and loss of antioxidant enzyme activity.

In this experimental study we investigated the protective effect of aqueous extract of *Podophyllum hexandrum *Free radicals e.g. superoxide radical, hydrogen peroxide and hydroxyl radical, from both endogenous and exogenous sources, are implicated in the etiology of several degenerative diseases, such as coronary artery disease, stroke, rheumatoid arthritis, diabetes and cancer [22]. High consumption of fruits and vegetables is associated with low risk for these diseases, which is attributed to the antioxidant vitamins and other phytochemicals [23,24]. The extent of initial damage caused by free radicals is further amplified by Fenton reaction generated hydroxyl radicals in the presence of superoxide and hydrogen peroxide [25]. Thus, the redox state and concentration of iron ions in the cellular milieu plays a crucial role in amplification of damage [26] as they interact with membranes to generate alkoxyl and peroxyl radicals, thereby inflicting further damage to the cellular system [27].

Superoxide is biologically important since it can be decomposed to form stronger oxidative species such as singlet oxygen and hydroxyl radicals, which are very harmful to the cellular components in a biological system [28]. Superoxide radical is generated from O_2 _by multiple pathways [29,30]. Using NBT assay system to generate superoxide radical, dose dependent inhibition was observed in the increasing concentration of *Podophyllum hexandrum *rhizome aqueous extract indicating its potential to possess scavenging properties.

Hydrogen peroxide itself is not very reactive, but it can give highly reactive species ^•^OH radical through Fenton reaction [31]. Earlier reports suggest that H_2_O_2 _could induce DNA break in the intact cell and purified DNA [32]. The H_2_O_2_-scavenging activity of *Podophyllum hexandrum *aqueous extract and the standard BHT increased in a dose dependent manner. With comparable results observed at highest concentration. Similar results were reported by Duh [33] for *Chrysanthemum morifolium *with high relationship between phenolic content and scavenging activity of the aqueous extracts on hydrogen peroxide. As previously reported by Chaudhary *et al.*, that *Podophyllum hexandrum *possess strong antioxidant activity against superoxide and hydroxyl radical under in vitro conditions [34]. Chawla *et al*., have also established the antioxidant potential of different extracts of *Podophyllum hexandrum *[35].

The level of kidney and lung MDA in CCl_4 _treated group was significantly higher than the control group. The increase in MDA level in both the tissues suggests enhanced peroxidation leading to tissue damage and failure of the antioxidant mechanisms to prevent the production of excessive free radicals. Our previous results have shown that ethanolic extract of *Podophyllum hexandrum *possess strong hepatoprotective activity against CCl_4 _induced damage in albino rats [36]. Similar results were previously reported in kidney by Ogeturk [37] and liver tissues by Yang [38] and Melin [39], which stated that CCl_4 _metabolized by cytochrome p-450 generates a highly reactive free radical, and initiates lipid peroxidation of the cell membrane of the endoplasmic reticulum and causes a chain reaction. These reactive oxygen species can cause oxidative damage in DNA, proteins and lipids. However pretreatment of *Podophyllum hexandrum *extract in this study significantly prevent CCl_4_-induced lipid peroxidation in kidney and lung tissue. Our results are in conformation to the already published report by Padma and Setty [40] that administration of aqueous extract of *Phyllanthus fraternus *significantly decreased the carbon tetrachloride induced lipid peroxidation in different organs of rats under *in vivo *conditions.

GSH as we know is involved in several defense processes against oxidative damage protects cells against free radicals, peroxides and other toxic compounds [41]. Indeed, glutathione depletion increases the sensitivity of cells to various aggressions and also has several metabolic effects. It is widely known that a deficiency of GSH within living organisms can lead to tissue disorder and injury [42]. In our study, the kidney and lung GSH level in CCl_4 _treated group was significantly decreased compared with control group. Likewise we [36] and others, Ohta [43], reported a significant decrease in the GSH content in different organs of rats, when injected with CCl_4_. Pretreatment however, with *Podophyllum hexandrum *aqueous extract increased GSH level as compared with CCl_4 _groups and thus affording protection. The antioxidant effects are likely to be mediated by the restoration of CCl_4 _induced decreased SOD, GR, GPx and GST activities in various tissues of rats. Treatment of rats with *Podophyllum hexandrum *aqueous extract significantly increased rat lung and kidney SOD, GR, GST and GPx activities. Tirkey [44] have recently conducted experiments to determine the effect of CCl_4 _on the renal damages in rats and obtained similar results. All these enzymes are major free radical scavenging enzymes that have shown to be reduced in a number of pathophysiological processes and diseases such as diabetes [45]. Thus, activation of these enzymes by the administration of *Podophyllum hexandrum *aqueous extract clearly shows that *Podophyllum *through its free radical scavenging activity could exert a beneficial action against pathophysiological alterations caused by the presence of superoxide, hydrogen peroxide and hydroxyl radicals.

## Conclusion

Combining all, we could conclude that the aqueous extract of *Podophyllum hexandrum *exhibits good antioxidant activity in both *in vitro *and *in vivo *experiments. *In vitro *antioxidant tests proved that the plant possesses components with strong superoxide and hydrogen peroxide radical scavenging activity. Study also suggests that the extract also possess potential to protect the kidney and lung tissue against oxidative damages and could be used as an effective protector against CCl_4 _induced kidney and lung damages. Further works are needed to fully characterize the active principles present in the plant responsible for these functions and elucidate its possible mode of action.

## Competing interests

The authors declare that they have no competing interests.

## Authors' contributions

SAG: Designed the study, conducted the experiments, analyzed the data and drafted the manuscript. EH and AH: made substantial contributions to the design of the study, the collection of the data as well as the interpretation and analysis of the data. They also drafted the manuscript and gave final approval for its submission to the Journal for consideration of publication. AM: made substantial contributions to the design of the study, the collection of the data as well as the interpretation and analysis of the data. He also drafted the manuscript and gave final approval for its submission to the Journal for consideration of publication. MAZ: the investigation-in-charge for the study, made substantial contributions to the design of the study, as well as the interpretation and analysis of the data. He also drafted the manuscript and gave final approval for its submission to the Journal for consideration of publication. YQ, ZM and BAZ: Made substantial contributions in the design of study and also helped in the compilation of the manuscript. All authors read and approved the final manuscript.

## Pre-publication history

The pre-publication history for this paper can be accessed here:

http://www.biomedcentral.com/1472-6882/11/17/prepub
